# Editorial: Using eco-friendly feedstuffs in ruminants to achieve a cleaner environment and reduced carbon footprint

**DOI:** 10.3389/fvets.2025.1681918

**Published:** 2025-08-29

**Authors:** Valiollah Palangi, Moyosore Joseph Adegbeye, Sadarman Sadarman

**Affiliations:** ^1^Department of Life Sciences, Western Caspian University, Baku, Azerbaijan; ^2^Research Centre for Animal Husbandry, National Research and Innovation Agency, Cibinong Science Centre, Bogor, Indonesia; ^3^Department of Animal Production and Health, Faculty of Agriculture, University of Africa, Toru-Orua, Bayelsa State, Nigeria; ^4^Department of Animal Science, Universitas Islam Negeri Sultan Syarif Kasim Riau, Pekanbaru, Indonesia

**Keywords:** cleaner environment, carbon footprint, eco-friendly, feedstuffs, livestock

## Introduction

The environmental impact of modern farming and the relentless drive for various extraction and production, often at the expense of replenishment and recycling, demand that we rethink our approach to livestock systems and nutrition. This rethinking must involve using alternative resources responsibly, promoting recycling, and reducing our carbon footprint. Given the growing urgency of this issue and the rapid expansion of research in this field, this editorial aims to highlight and discuss the latest findings, alongside alternative feed resources and additives that can be sustainably used for farm animals (1, 2). This Research Topic brings together contributions from around the world that focus on a common goal: adopting eco-friendly feed resources and approaches that improve or maintain animal performance while reducing environmental impacts, particularly greenhouse gas emissions. Collectively, these works reflect a shared vision for a sustainable, circular bio-economy in animal agriculture. The nine articles published (out of 11 submitted) span a broad range of innovative strategies, from valorizing agricultural by-products and developing functional feed additives, to exploring novel proteins derived from algae and insects. These papers came from geographic diverse sources with contributions from Egypt, Italy, South Korea, India, Turkey, China-Italy, Tunisia-Palestine, and two from China alone. The research covered a variety of animals including camels, sheep, cattle, pigs, insects, and even companion animals like dogs. All the papers are original research articles and employ diverse approach, including in vivo trials, *in-vitro* fermentation studies, and meta-analyses.

## Findings

### Valorizing wastes and agro-industrial by-products

The studies here were evaluated using *in vitro* means. For example, Ghazzawy et al. investigated the use of biochar derived from date palm (*Phoenix dactylifera*) seeds as a feed supplement for camels. Supplementation significantly enhanced fermentation parameters and reduced methane production *in vitro*. This shows the emerging role of biochar in not only soil amendment but also by-product and waste recycling (3), enteric emission mitigation, especially in arid regions where both camels and date palms are abundant. Vastolo et al. evaluated eight polyphenol-rich agro-industrial by-products, including grape, tomato, olive pomace, and hazelnut skin using sheep rumen fluid. By-products, from citrus and hazelnut by-products showed the greatest anti-methanogenic potential. Similarly, Ghzayel et al. examined carob leaves collected from Tunisia and Palestine that were treated with NaOH, urea, or polyethylene glycol. While treatment effects were highly dependent on the geographical origin of the leaves, the study confirmed the promise of agroforestry residues as a viable source of feed for ruminants, particularly in dryland ecosystems. These results promote the integration of regional waste streams into livestock diets as part of circular agricultural systems.

### Exploring novel protein sources: insects, algae, and fermented gases

The global race to identify viable and scalable alternative feed resources continues, especially in countries where governments recognize the importance of livestock farming, whether from a food security, economic, or environmental perspective. A comprehensive meta-analysis by Gao et al. explored the feasibility of using insect-derived meals, such as those from *Tenebrio molitor, Hermetia illucens*, and *Bombyx mori* in ruminant diets. The authors found that moderate inclusion ( ≤ 30%) of these high-protein feeds support digestibility and rumen fermentation, while also boosting growth performance in some trials. The oriental hornet (*Vespa orientalis*) was particularly promising, as it may hold untapped potential among underexplored insect species. Palangi et al. investigated the algae-nanoparticles relationship to assess the anti-methanogenic effect of *Chlamydomonas reinhardtii* combined with magnesium oxide and magnesium sulfide nanoparticles. I*n vitro* data showed significant improvements in gas production, digestibility, and volatile fatty acid profiles, pointing to the dual role of algae as a methane inhibitor and nutritional enhancer. While dogs may seem like an unusual inclusion in this Research Topic, it is important to understand that they are considered part of the broader category of farm animals, not necessarily as food animals (though this occurs in some cultures), but more commonly as companion animals. In a novel extension of microbial protein sources, Babu et al. conducted a pilot study on dogs using a fermented protein derived from methane gas. These alternative feeds imply the broader viability of insect and single-cell proteins as sustainable feed ingredients across species.

### Additives and dietary interventions for methane reduction

Methane mitigation via dietary supplements was also examined by Zhou et al., who evaluated the combined use of 3-nitrooxypropanol (NOP) and L-malate in dairy cows. The NOP supplementation alone reduced enteric methane by 54%, with no adverse effects on milk yield. When combined with L-malate, methane emissions were reduced by 51%, with added benefits to milk fat and protein composition. These results reinforce the importance of precise feed additives as tools for emission reduction without compromising productivity.

### Replacing conventional ingredients without sacrificing performance

The feasibility of replacing high-demand ingredients like soybean meal was addressed by Zhao et al., who demonstrated that mixed plant proteins, including rapeseed meal, palm kernel meal, and dried distillers grains with solubles (DDGS), could effectively substitute soybean meal in pig diets without negatively affecting growth or carcass quality. While not directly targeting methane reduction, these substitutions could contribute to feed sustainability and reduce deforestation-linked inputs. Meanwhile, Malik et al. conducted a meta-analysis on the effects of DDGS on methane emissions in cattle. Contrary to some expectations, DDGS had no significant effect on methane production or dry matter intake. However, its neutrality indicates that it can be used without exacerbating emissions, offering flexibility in diet formulation.

## Conclusion

These studies collectively illustrated the need to integrate diverse feed innovations including locally available by-products, novel proteins, targeted additives, and smart replacements to enable meaningful reductions in the carbon footprint of ruminant systems. This Research Topic also emphasizes the need for contextual evaluation as the effectiveness of feed interventions often depend on species, geography, processing methods, and dietary inclusion levels. For example, the performance of carob leaves or polyphenol-rich by-products was found to vary by origin and treatment method, and the efficacy of insect meals differed by species and inclusion rate. As global demand for animal protein rises, sustainable intensification must become a priority and feed innovations offer one of the most immediate levers for change.
